# Chewing Sugar-Free Gum Reduces Ileus After Cesarean Section in Nulliparous Women: A Randomized Clinical Trial

**DOI:** 10.5812/ircmj.6458

**Published:** 2013-04-05

**Authors:** Farideh Mohsenzadeh Ledari, Shanaz Barat, Mouloud Agajani Delavar, Seyed Zahra Banihosini, Soriya Khafri

**Affiliations:** 1 Department of Midwifery, Babol University of Medical Sciences, Babol, IR Iran; 2 Fatemeh Zahra Infertility and Reproductive Health Research Center, Babol University of Medical Sciences, Babol, IR Iran; 3 Fatemezahra Infertility and Reproductive Health Research Center, Department of Social Medicine, Babol University of Medical Sciences, Babol, IR Iran

**Keywords:** Chewing Gum, Ileus, Cesarean Section, Flatulence, Randomized Controlled Trials as Topic

## Abstract

**Background:**

Gum chewing after cesarean section may stimulate bowel motility and decrease duration of postoperative ileus.

**Objectives:**

The current study assessed the effect of chewing sugar-free gum on the return of bowel function, where cesarean section had been performed in nulliparous women.

**Materials and Methods:**

In a randomized clinical trial, 60 patients, scheduled for cesarean section were randomly divided in to 2 groups gum-chewing group (n = 30) and control group (n = 30) postoperatively. The patients in the gum-chewing group postoperatively chewed sugar free gum 3 times daily each time for 1 hour until discharge. The patients' demographic characteristics, duration of surgery, mean hunger time, flatus and bowel motility were compared in the two groups.

**Results:**

There was no significant difference between the 2 groups regarding patient demographics, intraoperative, and postoperative care. In the gum-chewing and the control group there was a significant difference in the mean postoperative interval of the first bowel movement (20.89 ± 8.8 versus 27.93 ± 9.3 hours, P = 0.004), the first feeling of hunger (10.37 ± 6.0 versus 16.33 ± 9.3 hours, P = 0.005), the first passage of flatus (25.02 ± 5.8 versus 31.08 ± 9.7 hours, P = 0.003), and the first defecation (31.17 ± 5.3versus 40.08 ± 8.8 hours, P = 0.000) respectively, which were significantly shorter in the gum-chewing group compared to those of the control group. There were no major complications in either group. All patients in the gum-chewing group tolerated it without any major complications and side effects.

**Conclusions:**

The study results demonstrated that bowel motility after cesarean section in nulliparous women can be accelerated by gum chewing which is a useful, inexpensive and well-tolerated method for mothers in post-cesarean section.

## 1. Background

Cesarean section is the most common surgery which is associated with postoperative changes in autonomic nervous system, leading to decreased bowel movements and driven problems (1). Ileus is referred to the delay, lasting for three to five days, in resumption of regular bowel movement following abdominal surgery (2), and is one of the major problems of post-abdominal surgery along with increased hospital stay, postoperative pain, abdominal distension, inability to start feeding, breastfeeding, and eventually delay in recovery (3). Ileus occurs in cases of opioid and drug interaction and abdominal surgery, especially in operations with excessive manipulation, and temporarily contributes to stop peristalsis (bowel movement); the related mechanism is probably dysfunction in parasympathetic system activity (inhibitory neurons) (4). Since, gynecology and obstetrics professionals have traditionally withheld postoperative oral intake to cesarean patients until the return of bowel function characterized by symptoms such as bowel movements, passage of flatus, defecation, and feeling of hunger (5), when the first passage flatus or stool is noted it shows an initial return of bowel function. Studies have demonstrated that early postoperative feeding could be safe prior to the return of flatus or stool (6), such a delay in the initiation of feeding eventuates in increased cell breakdown, delayed wound healing, elevated risk of infection and the need for more intravenous feeding, and eventually additional costs on healthcare system as well as the family (7). There is no specific treatment for postoperative ileus; however, several methods such as nasogastric suctioning, early feeding, intravenous fluid injection (7, 8), local analgesia, reducing intravenous drug consumption, minimal surgical manipulation, use of cyclooxygenase inhibitors, non-steroidal anti-inflammatory drugs, and drinks with high carbohydrate content (9) are recommended to decline the occurrence of postoperative ileus.

Gum chewing causes a person feel faint due to stomach stimulation and secretion of gastric and digestive juices, it provokes the person to eat and increases peristaltic bowel movements and hastens ileus recovery (10-13); it has also been recently considered by researchers as a strategy toward ileus reduction. In some studies, including Satij et al. (2006) and Maeboud et al. (2010), the beneficial effect of gum chewing has been approved in the resumption of bowel function (14, 15), but in some other studies such as Quah et al., contradictory findings have been achieved for the effects of gum chewing on peristaltic movements and digestive system stimulation (16). Gum-chewing has been studied over the last decade as a form of sham feeding to stimulate bowel recovery after surgery. The presumed mechanism of action is vagal cholinergic (parasympathetic) stimulation of the gastrointestinal tract, similar to oral intake but with theoretically less risk of vomiting and aspiration. In five such trials in patients undergoing colon resection, gum-chewing shortened the time until first flatus and bowel movement, but made no significant difference in the length of stay (17). At the very least, gum-chewing immediately after surgery is a cheap and harmless strategy to reduce postoperative ileus, and it might make the patient more comfortable however it seems that there is a necessity for more investigation on such a least-expensive physiological method in stimulating the return of bowel function.

## 2. Objectives

The current study was performed to investigate the effect of gum chewing on the return of intestinal function in women with cesarean section, so that a positive step can be taken toward diminishing their problems in fields of timely and early prevention of ileus.

## 3. Materials and Methods

The present randomized controlled clinical trial was conducted on 60 women participants at cesarean section with local anesthesia (spinal) in the gynecology ward of Rouhani Hospital of Babol during June 2010- March 2011. All patients with the same pre- and post-operative care underwent cesarean section (emergency, elective) by a transverse incision on the uterus and pfannenstiel incision on the abdomen by the same gynecology surgeon. Inclusion criteria consisted of cesarean delivery and consent for the study participation. Exclusion criteria included history of drug consumption, especially opioids, water and electrolyte disturbances, pancreatitis or peritonitis, history of abdominal surgery other than cesarean section, no willingness to cooperate, intra- and post-operative complications, inability to chew gum, withdrawal, diabetes, preeclampsia, ruptured water sac, hypothyroidism, and muscular and neurological disorders. The participants were given a thorough description on the research approach prior to entering the study. Giving consent, all patients kept their collaboration to the end of the study, and no case was excluded. A questionnaire which had been designed based on characteristics and objectives of the research, was first filled out for each participant and then the patients were divided into gum-chewing and the control group; the former was prescribed the sugar-free gum "Orbit" after recovery from anesthesia (6 h after surgery) three times a day, each time for one hour, until being discharged, and the latter received the ward's standard and routine care, so that they could be examined for the effects of gum chewing (sham feeding) alone. Data-collecting instruments included the interview form, questionnaires, and the examination of subjects. All patients were asked to note down the time to the first bowel movement, passage of flatus, defecation, and feeling of hunger. The research assistant who was unaware of gum prescription, visited the patients regularly, every one hour, and recorded the time of the first bowel movement, passage of flatus, defecation and feeling of hunger separately in data-collecting forms. The obtained data were analyzed by SPSS16 statistical software using t-test and chi square, and P < 0.05 with 95% confidence interval was considered as the statistically significant level.

## 4. Results

The mean age of participants was 25.10 years. There was no statistically significant difference between the two groups in terms of demographic characteristics such as age, body mass index, parity, duration of surgery, number of miscarriages, curettages, time to the first feeding, the amount of serum intake, and type of cesarean section: emergency and elective were matched (Table 1). Type of cesarean section: emergency and elective, had no effect on the return of bowel function (Table 2). The mean time to the first bowel movement was 20.89 hours in gum-chewing and 27.93 hours in the control group (P = 0.004). The mean time to the first defecation was 31.17 hours in the gum-chewing and 40.08 hours in the control group (P = 0.000). The mean time to the first passage of flatus was 25.02 hours and 31.08 hours in gum-chewing and the control group respectively (P = 0.003), and the mean time to the onset of hunger was 10.37 hours in the gum-chewing and 16.33 hours in the control group (P = 0.005). None of the participants felt dissatisfied with chewing gum and none were excluded from the study (Table 3).

**Table 1.** Participants

Variables	Gum-chewing, Mean ± SD	Control, Mean ± SD	P value
**Age(year)**	25.00 ± 6.02	25.20 ± 6.55	0.09
**Body mass index**	30.33 ± 5.1	31.94 ± 4.58	0.20
**Duration of surgery(min)**	32.06 ± 8.1	30.50 ± 4.4	0.36
**Feeding time(hour)**	21.69 ± 2.02	22.73 ± 4.17	0.23
**The amount of serum intake(liter)**	2.93± 0.37	2.80 ± 0.61	0.32

**Table 2.** Resumption of Bowel Function Following the Operation in Emergency and Elective

Variables	Emergency, Mean ± SD (n = 25)	Elective, Mean ± SD (n = 35)	P value
**Onset of bowel movement (hour)**	24.92 ±8.87	24.13 ±10.19	0.75
**Onset of defection(hour)**	36.16 ±7.54	35.13 ±9.29	0.65
**Onset of gas passage(hour)**	29.40 ±7.44	27.00±8.24	0.25

**Table 3.** Resumption of Bowel Function Following the Operation in Both Groups (n=60)

Variables	Gum-chewing, Mean ± SD	Control, Mean ± SD	P value
**Onset of bowel movement(hour)**	20.89 ± 8.8	27.93 ± 9.3	0.004
**Onset of defecation(hour)**	31.17 ± 5.3	40.08 ± 8.8	0.000
**Onset of gas passage(hour)**	25.02 ± 5.8	31.08 ± 9.7	0.003
**Feeling of hunger(hour)**	10.37 ± 6.0	16.33 ± 9.3	0.004

## 5. Discussion

The study findings demonstrated no significant difference in terms of the mean age, duration of surgery, BMI, time to start oral feeding, serum intake, number of pregnancies, miscarriages, curettages and types of cesarean section between the control and sugarless gum chewing group of patients; the latter were well tolerating the gum and showed no feeling of dissatisfaction, and none were therefore excluded from the study; likewise, in Shang, Ghafouri, Yaghmaei and Safdari studies, the two groups of gum-chewing and control had no significant difference on demographic variables and other mentioned characteristics (2, 5, 6, 17); however, the results of the present research showed a time reduction to the first bowel movement, defecation, passage of flatus, and feeling of hunger following gum chewing after the cesarean section. In the current study, the mean time to the first passage of flatus revealed remarkable difference between the two groups, which is the same as Yaghmaei’s study reporting a comparison of oral intake profiles at 2 and 8 hours following elective and emergency cesarean section under spinal anesthesia on 112 women in Zahedan in 2009, (7.75 ± 2.18 h and 13.18 ± 2.18 h P = 0.03) (5). In the current study, the mean time to the first bowel movement revealed remarkable difference between the two groups, which is in accordance with Safdari study on the effects of gum chewing after elective cesarean section on 120 primiparous women in Shahrekord in 2011, reporting 7.4 ± 1.71 h and 15.7 ± 3.44 has the mean time to the first bowel movement in the gum chewing and the control group respectively (6); the finding is also consistent with Schuster’s study on the impact of gum chewing after sigmoid-colectomy surgery on 34 patients in 2006, in which the mean time to the first bowel movement was 63.2 ± 5.4 h and 89.4 ± 2.4 h in the gum chewing and the control group respectively (11). Nonetheless, in Akhlaghi`s research on the effect of gum chewing on the resumption of bowel function after cesarean section on 120 patients in Mashhad in 2008, the two groups were different in terms of the feeling of bowel movement, but the difference was not statistically significant, as the mean difference was 14.7 ± 6.5 h and 16.6 ± 8.4 h in gum-chewing and the control group respectively. This study is not in agreement with the current research, and the reason of the contradiction may be due to sampling and/or surgical conditions (3). Another variable examined in terms of bowel function was the feeling of hunger which was 3 hours earlier in the gum chewing group than the other one, which was statistically significant; this finding is in consensus with Satij’s results following the cesarean section in 2006 (14); however, in Schuster’s study, the two groups were different on the feeling of hunger, but the difference was not statistically significant, as the mean time to the feeling of hunger was 63.5 ± 10.4 h and 72.8 ± 31.1 h in the gum chewing and the control group respectively (11); such a discrepancy could be owing to the small sample size in Schuster’s study. In the current research, there was a significant difference between the two groups regarding the mean time of the first defecation as it happened 8 hours earlier in the gum-chewing than the control group, similar to the results obtained by Maeboud, Ghafouri, Hirayama, Hocevar and Abdollahi (1, 2, 15, 18, 19). In Maeboud’s study on 200 patients after elective cesarean section in Egypt in 2010, the mean time of defecation was 21.1 ± 4.7 h and 30.00 ± 8.2 h earlier in gum-chewing and the control group, and in Hirayamai and Ghafouri`s studies, respectively on colorectal surgery on 22 patients in 2006 in Japan, and 50 patients with upper gastrointestinal tract surgery in 2008 in Tehran, defecation time was 15 hours earlier in the gum-chewing than the control group in both studies, which was statistically significant. In Abdollah investigation on 46 patients following appendectomy surgery in Gorgan in 2011, time of the first defecation was 24 h earlier in the gum-chewing than the control group which was statistically significant. However, in Quah research on 38 patients after left colon cancer surgery in England in 2006, no statistical difference was observed in the time of the first defecation between the gum chewing (3.2 ± 1.5) and the control (3.9 ± 1.5) group (16), which the small sample size and type of surgery may be the reasons for such a difference. The mean passage of flatus was the other variable evaluated on intestinal function, happening, an average of 5 hours earlier in the gum chewing than the control group; this finding is consistent with Kouba’s investigation on 102 patients undergoing bladder radical surgery in 2007 in America, in which the time of the first passage of flatus was respectively 2.4 and 2.9 days in the gum-chewing and the control group, showing acceleration of gas passage following chewing gum after bladder surgery (20). In Ngowe’s study in 2010 on 46 patients with open appendectomy, the mean time of gas passage was 2.2 and 3.0 days in the gum-chewing and the control group (21). In Choi’s study on 60 patients in 2011, the mean time to the passage of flatus was 60 h and 48 h in gum-chewing and the control group which the difference was statistically significant (22); whilst, Quah reported no remarkable difference between the two groups in terms of gas passage. There is not yet an independent investigation on the exact gum chewing mechanism of action, however, some theories discuss chewing as a form of sham feeding that stimulates food digestion and secretion of salivary and hepatic glands through the vagus nerve stimulation and increases the plasma concentration of gastrin, neurotensin, pancreatic polypeptide, and duodenal alkaline secretion. Thus, gum chewing directly augments intestinal stimulation through gastrointestinal releasing hormones and increasing saliva and pancreatic juices and subsequently promotes ileus recovery (23, 24).

The results of the current study indicated that gum chewing following cesarean section is accompanied by reduction in the time of the passage of flatus, bowel movements, and feeling of hunger, and no complication has been reported in this regard; moreover, it can be added to post- cesarean care without any concern on early post-operation feeding as a low-cost, safe and tolerable treatment in early intestinal stimulation to reduce ileus associated complications. Therefore, it is recommended that the effect of chewing gum be investigated on ileus prevention in other operations such as hysterectomy. Further investigations with a large sample size are needed to define the effect of gum chewing on length of hospital stay in emergency and elective cesarean separately.
